# Supplementary material for: Perinatal S-Adenosylmethionine Supplementation Represses PSEN1 Expression by the Cellular Epigenetic Memory of CpG and Non-CpG Methylation in Adult TgCRD8 Mice
Source: Int J Mol Sci. 2023 Jul 19;24(14):11675. doi: 10.3390/ijms241411675 (PMC10380323; doi:10.3390/ijms241411675)
Supplement: Supplementary file 1 [file ijms-24-11675-s001.zip › Figure S1.pdf]

Suppl. Fig. S1: PSEN1 methylation in WT mice

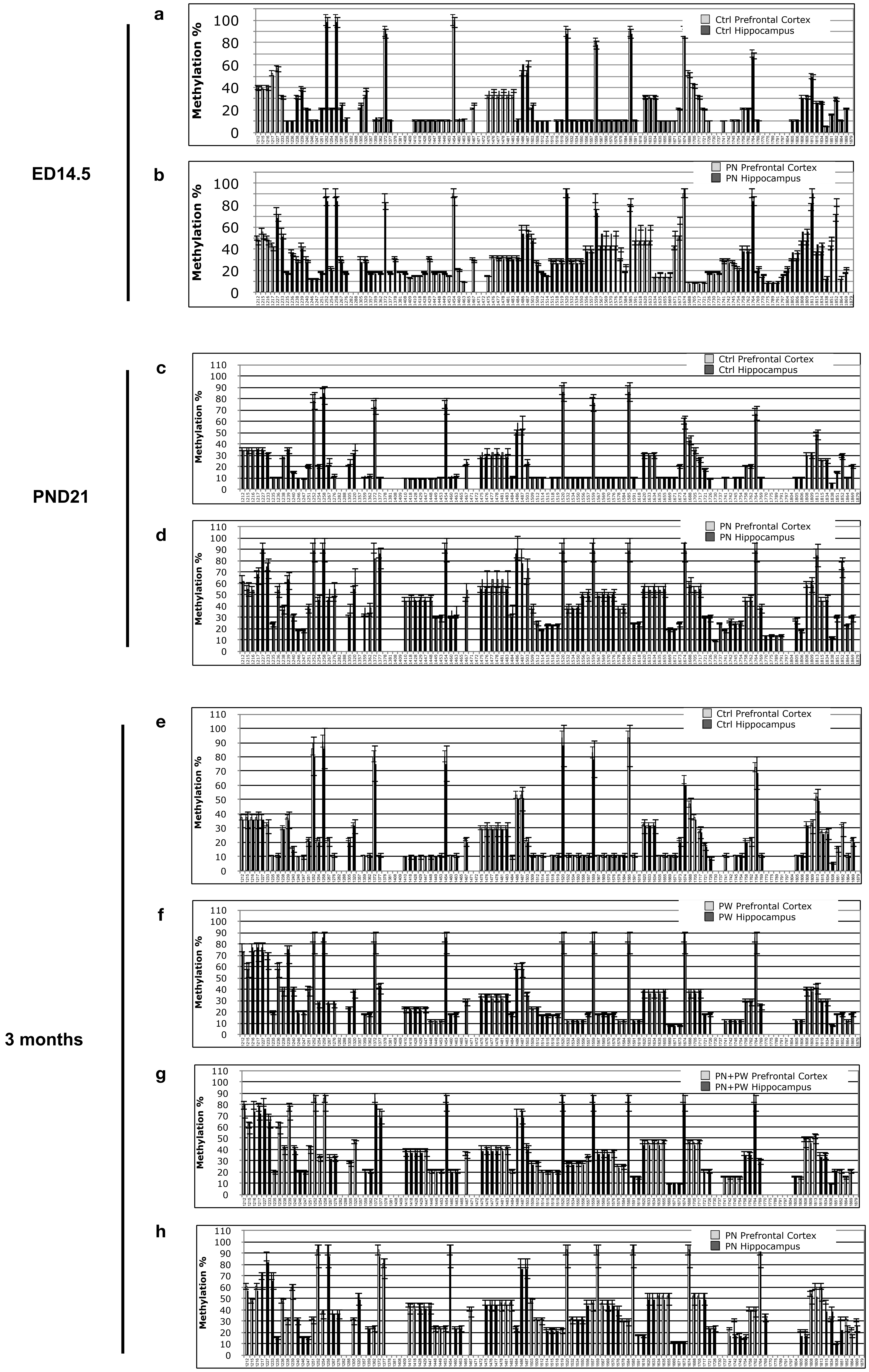

Supplementary Figure S1: PSEN1 promoter CpG and non-CpG methylation patterns in WT mice brain.

a) Ctrl diet and b) perinatally SAM-supplemented at ED14.5; c) Ctrl diet and d) perinatally SAM-supplemented at PND21; e) Ctrl diet, f) post-weaning SAM-supplemented diet, g) perinatally + post-weaning SAM-supplemented diet, h) perinatally SAM-supplemented diet at 3 months; N=10. Histograms show methylation % (± standard deviation, Y-axis) of each cytosine; labels on X-axis indicate the cytosine position on the reference sequence from the 5' (left) to the 3' (right) of the promoter, i.e. with the region proximal to the Transcription Start Site (on the right). Light grey columns represent methylation in Prefrontal cortex, dark grey columns represent methylation in Hippocampus.
